# Virtual screening, *in silico* pharmacokinetic and toxicity profiling of colchicine-based inhibitors of estrogen receptor of breast cancer

**DOI:** 10.1016/j.toxrep.2025.101926

**Published:** 2025-01-25

**Authors:** Philip John Ameji, Amneh Shtaiwi, Rohana Adnan

**Affiliations:** a School of Chemical Sciences, Universiti Sains Malaysia, 11800, Penang, Malaysia; b Department of Chemistry, Federal University Lokoja, P.M.B. 1154, Lokoja, Nigeria; c Chemistry Department, Faculty of Science, Applied Science Private University, Amman 11931, Jordan

**Keywords:** Breast cancer, Virtual screening, Toxicity, MM/GBSA, DFT, Pharmacokinetics

## Abstract

The declining efficacies of existing drugs against estrogen receptor positive (ER+) breast cancer due to multidrug resistance, acute toxicities, and poor pharmacokinetic properties has necessitated the discovery of newer ones. In this study, colchicine analogues with proven *in vitro* activities against breast cancer cells were screened against estrogen receptor alpha (ERα) *via* molecular docking simulations to identify some promising drug candidates. The identified ligands were further subjected to MM/GBSA calculations to ascertain their solvation-dependent Gibb’s free energy of binding (∆G_B_). Three most promising ligands (MPLs); 12, 16, and 21 with ∆G_B_ values of − 40.37, − 40.31, and − 40.26 kcal/mol, respectively, were identified. When compared with tamoxifen (standard drug) whose ∆G_B_ value is − 38.66 kcal/mol, the MPLs appear more potent. The kinetic stabilities of 12, 16, and 21 were confirmed by DFT (B3LYP/6-31G*) calculations and the time-dependent thermodynamic stabilities of their complexes with ERα were established by molecular dynamic simulations. In addition, the MPLs display positive pharmacokinetic and toxicity profiles and could be excellent sources of potent and non-toxic drug candidates against ER+ breast carcinoma.

## Highlights

• Some potent and non-toxic colchicine-based inhibitors of estrogen receptor (ER) of breast cancer have been identified.
• The new anti-cancer agents appear more potent and efficacious than Tamoxifen.
• The kinetic stability of the drug candidates has been established by DFT calculations.
• Coarse-grained molecular dynamic simulation supports the time-dependent thermodynamic stability of the ligand-ER complexes.

## Graphical Abstract

**Figure:**
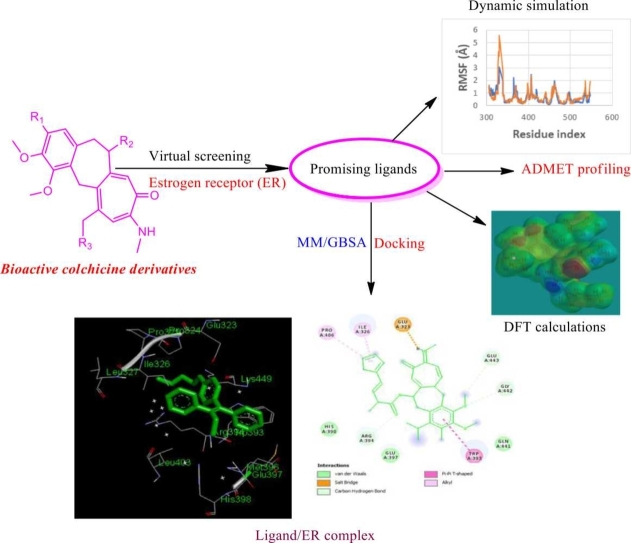

## 1 Introduction

Breast cancer is the predominant cancer type in women and about 12 % of the female population are affected in their lifetime [1]. This type of cancer occurs when there is a change in the molecular features of normal cells of the breast that culminates into uncontrollable growth and proliferation of the cells [2]. Statistically, about 2.3 million cases and 685,000 deaths due to breast cancer were recorded globally and it has been projected that the mortality rate may approach 4.4 million by the year 2070 [3]. Obesity, sedentary lifestyle, early menarche, use of hormonal therapy and heredity are some of the major risk factors of the disease [4], [5].

Among the various breast cancer types, estrogen receptor positive (ER+) is the most common and it accounts for over 80 % cases of breast carcinoma in women [6], [7]. In this type of breast cancer, proteins known as estrogen receptors (ER) on the cancerous cells provide binding surfaces to estrogen hormones which in turns provides growth signals to the cells [2]. Thus, over-expression of estrogen receptors has been identified as a major prognostic and diagnostic biomarker of ER+ breast carcinoma and inhibition of ER is considered a gold standard in ER+ breast cancer treatment [8]. The US FDA approved inhibitors of ER include tamoxifen, raloxifene, toremifene, fulvestrant, anastrozole and letrozole but their efficacies are declining over the years due to recorded instances of rising drug resistance and their poor pharmacokinetic and toxicity profiles [7], [9], [10], [11], [12], [13]. Thus, it has been become expedient to search for newer and better alternatives.

The capital intensiveness and enormous time requirement that characterizes traditional drug discovery processes have constituted serious bottlenecks to the identification of novel drugs against ER+ breast cancer. However, the application of *in silico* techniques have helped to ameliorate the aforementioned challenges through the provision of economical, greener and faster drug discovery strategies. In recent times, some efforts have been made by researchers towards discovering new inhibitors of ER *via* computer-aided drug discovery approaches [14], [15], [16], [17], [18], [19], [20], [21]. In this study however, colchicine amides and sulfonamides with proven antiproliferative properties against human breast cells (MCF-7) were screened against ER *via* molecular docking simulations and molecular mechanics, general born surface area (MM/GBSA) calculations to ascertain the strength and mechanisms of their binding interactions with the target receptor. The stability of the promising ligands and their complexes with ER was investigated *via* density functional theory (DFT) studies and molecular dynamic simulations, respectively. The fates of the ligands with particular emphasis on their possible absorption, distribution, metabolism, excretion and toxicity were investigated through pharmacokinetic and toxicity profiling.

## 2 Methods

### 2.1 Virtual screening

A data set of twenty-one (21) colchicine amides and sulfonamides (Table 1) with established *in vitro* anti-proliferative properties against MCF-7 cells were obtained from literature [22]. ChemDraw Ultra 12.0 was used to draw and assign proper 2D orientation to the ligands and the energy of each molecule was minimized using the semi-empirical method (Pm3) in Spartan 14’ v1.1.4 interface [23] and the AutoDock 4.2 tool interface was used to assign Gasteiger charges to the optimized ligands. Also, pdb file of ER target (PDB code: 5gs4) was downloaded from https://www.rcsb.org/structure/5GS4 and the co-crystallized ligand, water, and heteroatom attached to the macromolecule were removed using the Biovia Discovery Studio v6.1.0.15350 interface [24]. Protonation and addition of Kollman charges to the macromolecule was performed AutoDock 4.2 interface. Subsequently, EasyDock Vina 2.2, a graphical user interface of AutoDock Vina was used to dock the ligands into the active sites of ER protein target [25] by setting the centre of the grid box to; x = -12.267 Å, y = -10.250 Å, z = 6.4695 Å and dimension to x = 57.9185 Å, y = 46.8256 Å, z = 44.3671 Å. In the course of the docking calculations, nine conformations were considered for each ligand and the ones with the most favorable Gibb’s binding free energy were adopted as the best docking score. Visualization of the ligand-receptor interactions were done using the Discovery studio v6.1.0.15350 [26].

**Table 1:** Structure and *in vitro* anti-breast cancer activities of the compounds.

### 2.2 Oral bioavailability estimation, pharmacokinetic and toxicity profiling

Oral bioavailability describes the possibility of a therapeutic molecule to be administered *via* the oral route. The oral bioavailability potentials of the ligands were assessed using the Lipinski’s rule of five (RoF) which posits that a drug with molecular weight of less than 500 g/mol, less than 5 hydrogen bond donors (HBD), less than 10 hydrogen bond acceptors (HBA), and LogP value that is less than 5 would most likely be orally bioavailable [27]. In addition to the Lipinski’s rule, the oral bioavailability tendencies of the ligands were evaluated using the Veber’s rule that states that an orally bioavailable drug would have topological polar surface area that is less than 140 Å^2^ and rotatable bonds that are less than 10 in number [28]. The aforementioned physicochemical properties of the ligands as well as their pharmacokinetic and toxicity profiles were computed using the Deep-PK server at https://biosig.lab.uq.edu.au/deeppk/prediction.

### 2.3 MM/GBSA calculations

A cardinal objective of modern drug discovery is to find new therapeutic molecule that binds strongly to a target macromolecule. While this information could be obtained from molecular docking simulation, it does not put into consideration the effect of solvation on ligand-protein binding interaction. A more accurate method that address this loophole is the MM/GBSA calculations which computes the Gibb’s free energy change of binding interaction of a ligand to a macromolecule according to Eq. (1) [29].

(1) $$\Delta G=\;{∆H-T∆S=∆E}_{\mathrm{MM}}+\;{∆G}_{\mathrm{GB}}+\;{∆G}_{\mathrm{SA}}-T∆S$$

Where ∆E_MM_ is the sum of internal energy, van der Waals and electrostatic interactions; ΔG_GB_ is the polar contributions to solvation energy; ΔG_SA_ is the non-polar contribution to solvation energy, and TΔS is the change in conformational entropy upon ligand–protein binding interaction [29], [30]. The MM/GBSA-derived ∆G values of the ligands to ER were calculated with the aid of fastDRH server at http://cadd.zju.edu.cn/fastdrh/ [31].

### 2.4 Coarse-grained molecular dynamic simulation

The time-dependent behavior of the complexes of the ligands with ER was investigated using the coarse-grained (CG) molecular dynamic (MD) simulations. The coarse-grained MD simulation approach is a faster and more efficient alternative to the classical all-atom MD performed at shorter time-scale which might not be sufficient to cover the time scale of biological events that naturally occur at much longer periods [32], [33]. The CG MD was performed on the pdb files of the ligand-ER complexes with the aid of CABSflex 2.0 server hosted by the National Science Center of Poland at http://biocomp.chem.uw.edu.pl/CABSflex2/job/746d8804a364dc5/ [31], [32], [33].

### 2.5 Density functional theory calculation

The Density Functional Theory (DFT) provides a faster and cheaper approach for solving the nonrelativistic, time-independent Schrodinger equation. This theory postulates that the ground state energy and other molecular properties of a system are exclusive reserve of the system’s electronic density as shown in Eq. (2) [34].

(2) $${\;E}_{0}[\rho ]\;=\;T[\rho ]\;+\;V[\rho ]\;+\mathrm{Ec}[\rho ]$$

Where ρ is the electron density, E_0_ the ground state energy, T[ρ] is the kinetic energy component of the system, V[ρ] is the potential energy component comprising of coulomb interaction among the electron distributions and nuclei-electron interaction and (Ec[ρ]) is the exchange correlation energy [35]. The DFT (B3LYP/6–31 G*) method in Spartan ’14 v1.1.4 software was used to calculate the following electronic descriptors of the ligands; energy of HOMO (E_H_), energy of LUMO (E_L_), HOMO-LUMO energy gap (∆E) defined in Eq. (3), and global electrophilicity index (ω) defined in Eq. (4).

(3) ∆E = E_L_ – E_H_

(4) $$\omega =\;\frac{u^{2}}{2\eta }$$

Where μ is the electronic chemical potential defined in Eq. (5) and η is the chemical hardness defined in Eq. (6). Also, electronegativity (χ) and chemical softness (S) of the ligands were calculated with the aid of (7), (8), respectively.

(5) $$\mu =\;\frac{E_{L}+E_{H}}{2}\;=\;\frac{-(I+A)}{2}$$

(6) $$\eta =\;\frac{∆E}{2}\;=\;\frac{\left(I-A\right)}{2}$$

(7) $$\chi =\;\frac{\left(A+I\right)}{2}$$

(8) $$S=\;\frac{1}{2ƞ}$$

Where A and I are the electron affinity and ionization potential, respectively. According to Koopman’s theorem, A is the negative of LUMO energy while I is the negative of HOMO energy [36].

## 3 Results

### 3.1 Molecular docking based virtual screening and MM/GBSA studies

**Fig. 1:**
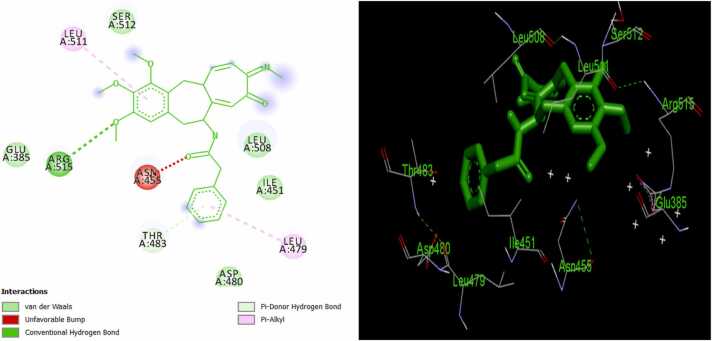
12/ER complex in 2D (left) and 3D (right).

**Fig. 2:**
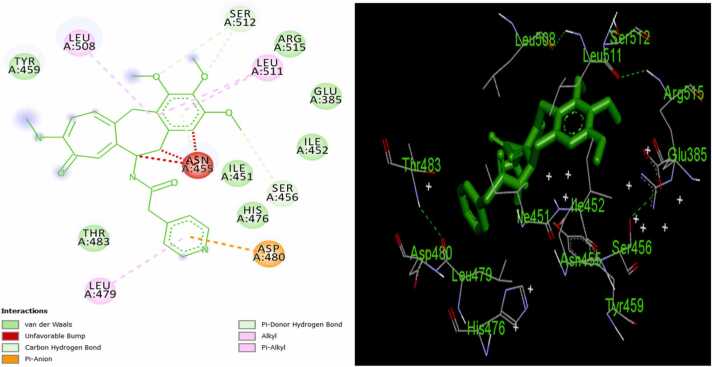
16/ER complex in 2D (left) and 3D (right).

**Fig. 3:**
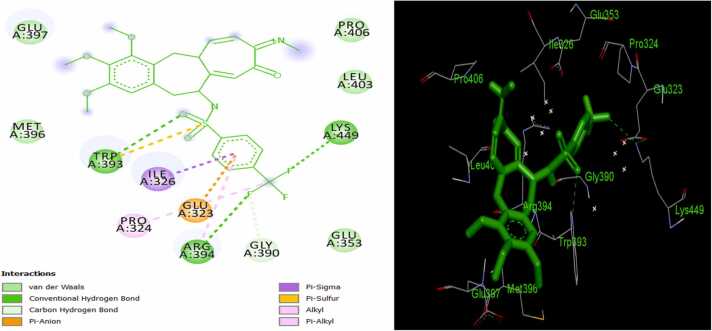
21/ER complex in 2D (left) and 3D (right).

**Fig. 4:**
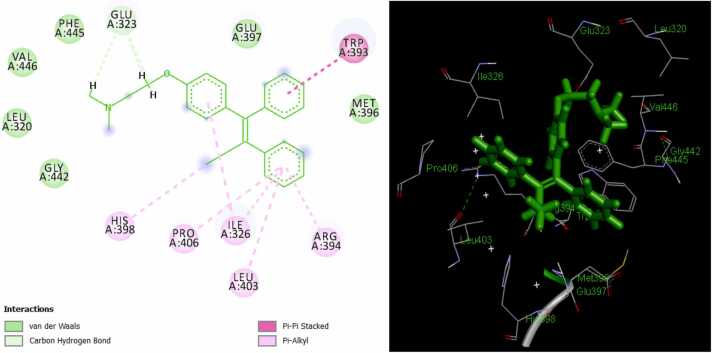
Ta/ER complex in 2D (left) and 3D (right).

**Fig. 5:**
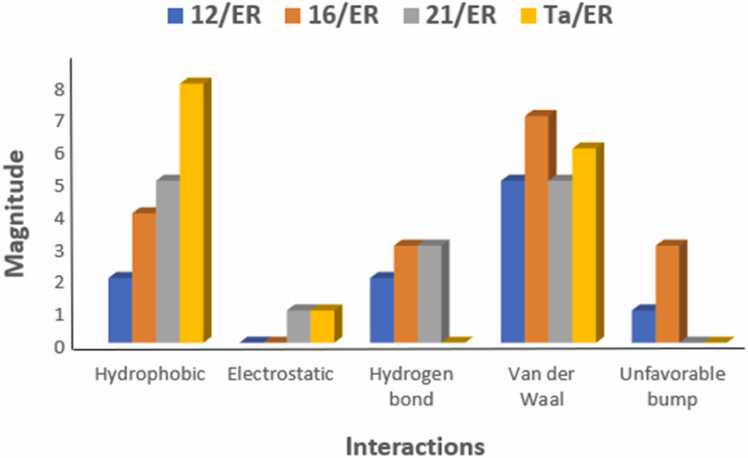
Mechanism of interactions in the complexes of the MPLs with ER target.

Molecular docking simulations are performed to ascertain the strength and mechanism of binding interaction of ligands to a target macromolecule while MM/GBSA provides a more physical interpretation of ligand-receptor binding interactions by putting into cognizance both bound and unbound states in addition to implicit solvation. The results of molecular docking and MM/GBSA studies on the binding interaction of the bioactive colchicine derivatives and ER are presented in Table 1, Table 2, Table 3, respectively. Also, the mechanistic details into the binding interactions in the complexes of the MPLs and the standard drug with ER are presented in Figs. 1–4. Furthermore, Fig. 5 showed the various types of interactions in the complexes.

**Table 2:** Average docking score of the ligands.

Complex	G_D1_ (kcal/mol)	G_D2_ (kcal/mol)	∆G_D3_ (kcal/mol)	∆G_D_ (kcal/mol)
1/ERα	− 6.6	− 6.6	− 6.6	− 6.6
2/ERα	− 6.8	− 6.8	− 6.8	− 6.8
3/ERα	− 6.1	− 6.0	− 6.2	− 6.1
4/ERα	− 6.9	− 6.9	− 6.9	− 6.9
5/ERα	− 6.7	− 6.7	− 6.6	− 6.7
6/ERα	− 6.5	− 6.5	− 6.7	− 6.6
7/ERα	− 6.5	− 5.2	− 5.2	− 5.6
8/ERα	− 6.9	− 6.9	− 6.8	− 6.9
9/ERα	− 6.5	− 6.5	− 6.5	− 6.5
10/ERα	− 6.9	− 6.9	− 6.9	− 6.9
11/ERα	− 6.2	− 6.6	− 6.2	− 6.3
12/ERα	− 6.9	− 7.0	− 7.0	− 7.0
13/ERα	− 7.5	− 7.5	− 7.5	− 7.5
14/ERα	− 7.0	− 7.9	− 6.9	− 7.3
15/ERα	− 6.3	− 6.3	− 6.5	− 6.4
16/ERα	− 7.0	− 7.0	− 7.0	− 7.0
17/ERα	− 6.3	− 6.3	− 6.3	− 6.3
18/ERα	− 6.5	− 6.6	− 6.6	− 6.6
19/ERα	− 5.9	− 5.9	− 5.9	− 5.9
20/ERα	− 6.2	− 6.0	− 6.6	− 6.3
21/ERα	− 7.6	− 7.7	− 7.6	− 7.6
Ta/ERα	− 6.3	− 6.2	− 6.2	− 6.2

G_D1:_ 1st docking score reading, G_D2:_ 2nd docking score reading, G_D3:_ 3rd docking score reading, ∆G_D_: average docking score.

**Table 3:** MM/GBSA based binding energy values of the promising ligands.

Complex	∆E_MM_ (kcal/mol)	∆G_GB_ (kcal/mol)	∆G_SA_ (kcal/mol)	∆G_B_ (kcal/mol)
4/ERα	− 39.11	− 4.63	7.26	− 36.48
8/ERα	− 42.30	− 4.95	8.35	− 38.9
10/ERα	− 41.52	− 4.74	6.90	− 39.36
12/ERα	− 44.4	− 5.22	9.25	− 40.37
13/ERα	− 41.28	− 4.51	8.03	− 37.76
14/ERα	− 35.80	− 4.46	9.24	− 31.02
16/ERα	− 44.20	− 5.17	9.06	− 40.31
21/ERα	− 46.99	− 5.8	12.58	− 40.26
Ta/ERα	− 44.47	− 5.47	11.27	− 38.66

∆E_MM_ = changes in the gas-phase molecular mechanics (MM) energy; ∆G_GB_ = Polar contribution to Δ*G*_solvation_; ∆G_SA_ = non-polar contribution to Δ*G*_solvation_; Tm = template ligand; ∆G_D_ = docking score; ∆G_B_ = estimated binding energy.

### 3.2 Oral bioavailability and ADMET profiles of the ligands

Oral bioavailability assessment is done to ascertain the suitability of a drug-like molecule to be administered *via* the oral route while ADMET investigations are performed to evaluate the pharmacokinetic and toxicity profiles of therapeutic molecules. Table 4, Table 5 present the ligands’ oral bioavailability and pharmacokinetic profiles, respectively, while their toxicity profiles are presented in Table 6.

**Table 4:** Computed descriptors of oral bioavailability of the Ligands.

Ligand	#HD	#HA	Mw (g/mol)	LogP	TPSA (Å^2^)	#RB
4	2	5	412.48	2.75	85.89	7
8	2	5	440.53	3.24	85.89	8
10	2	5	467.35	3.04	85.89	7
12	2	7	474.56	3.66	85.89	8
13	2	7	509.00	4.32	85.89	8
14	2	7	478.52	3.87	85.89	7
16	2	8	475.55	3.06	98.78	8
21	2	8	564.58	4.3	102.96	8
Ta	0	2	371.51	5.99	12.47	8

#HD; number of hydrogen bond donor, #HA; number of hydrogen bond acceptor, #RB; number of rotatable bonds, Ta; tamoxifen.

**Table 5:** Pharmacokinetics profiles of the ligands.

Ligand	GIA	P-gp^+^	VDss (logVDss)	BBB+	CYP2D6 substrate	CYP3A4 substrate	Clearance (log ml/min/kg)
4	Yes	Yes	0.70	No	Yes	No	4.68
8	Yes	Yes	1.18	No	Yes	No	8.40
10	Yes	Yes	0.73	No	Yes	No	6.02
12	Yes	Yes	0.40	No	Yes	No	9.26
13	Yes	No	1.35	No	Yes	No	8.61
14	Yes	Yes	1.51	Yes	Yes	No	7.74
16	Yes	Yes	0.93	No	Yes	No	5.22
21	Yes	No	2.91	Yes	Yes	No	7.18
Ta	Yes	Yes	4.47	No	Yes	Yes	10.36

GIA;gastrointestinal absorption, VDss; volume of distribution at steady state, P-gp+; P-glycoprotein substrate, BBB+; blood brain permeant.

**Table 6:** Toxicity profiles of the ligands.

Ligand	AMES mutagenesis	Biodegradation	Carcinogenesis	Liver injury 1	Reproductive effective
4	Safe	Safe	Safe	Safe	None
8	Safe	Safe	Safe	Safe	None
10	Toxic	Safe	Safe	safe	High
12	Safe	Safe	Safe	Safe	None
13	Safe	Safe	Safe	Safe	None
14	Safe	Safe	Safe	Toxic	None
16	Safe	Safe	Safe	Safe	None
21	Safe	Safe	Safe	Safe	None
Ta	Safe	Safe	Toxic	Safe	High

### 3.3 Molecular dynamic simulations

**Fig. 6:**
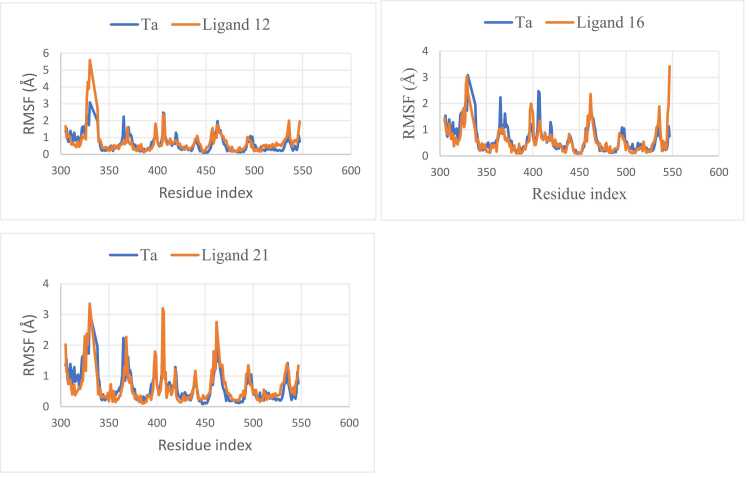
Coarse-grained molecular dynamic simulations of the complexes of the MPLs with ER.

A crucial requirement of a drug against ER+ breast carcinoma is its ability to form a stable complex with ER for a reasonable length of time. This information cannot be sufficiently obtained from molecular docking and MM/GBSA calculations. Thus, a recourse to molecular dynamic (MD) simulation studies. The results of the molecular dynamic simulations on the complexes of the MPLs and Ta with the receptor are presented in Fig. 6.

### 3.4 DFT studies

**Fig. 7:**
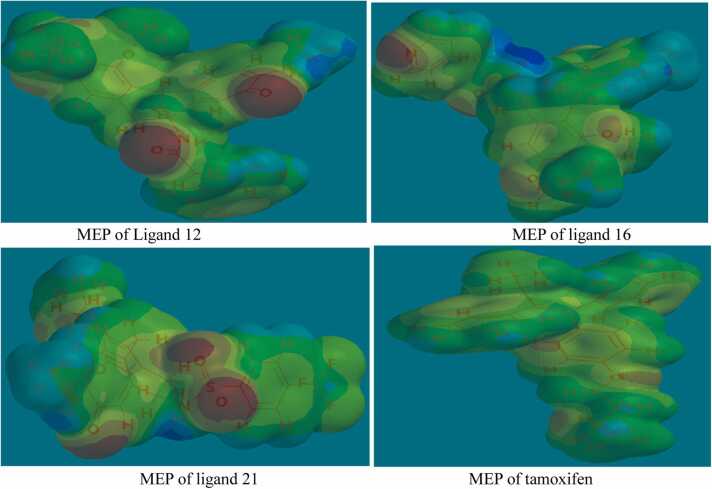
Electrostatic potential map of the most promising ligands and Ta Red color: most negative potential; blue color: most positive potential.

A crucial requirement of an ideal drug is its ability resist chemical and physical degradation within the boundaries of specified standard conditions in line with quality control specifications [42], [43]. The stability of molecules could be ascertained from the knowledge of their quantum mechanics-derived electronic descriptors. The reactivity descriptors of the MPLs and the reference drug are presented in Table 7 and their molecular electrostatic potential maps are presented in Fig. 7.

**Table 7:** Local and global reactivity descriptors of the most promising ligands.

**ligand**	**E**_**L**_**(eV)**	**E**_**H**_**(eV)**	**A (eV)**	**I (eV)**	**Χ (eV)**	**∆E (eV)**	**μ (eV)**	**η (eV)**	**S (eV)**	**ω (eV)**
12	− 1.51	− 5.29	1.51	5.29	3.40	3.78	− 3.40	1.89	0.26	3.06
16	− 1.66	− 5.42	1.66	5.42	3.54	3.76	− 3.54	1.88	0.27	3.33
21	− 1.56	− 5.22	1.56	5.22	3.39	3.66	− 3.39	1.83	0.27	3.14
Ta	− 1.24	− 5.75	1.24	5.75	3.50	4.51	− 3.50	2.26	0.22	2.71

## 4 Discussion

Drugs such as selective estrogen receptor modulators (SERMs) and selective estrogen receptor degraders (SERDs) used for the treatment of ER+ breast carcinoma function by binding strongly to ER and limits the affinity of estrogen hormones for ER surface. The outcome of molecular docking studies on the investigated colchicine analogues shown in Table 2 reveals that all the ligands bind spontaneously to the active sites of the receptor with ∆G_D_ values that range from − 5.6 to − 7.6 kcal/mol. However, ligands 4, 8, 10, 12, 13, 14, 16, and 21 with superior ∆G_D_ values of − 6.9, − 6.9, − 6.9, − 7.0, − 7.5, − 7.3, − 7.0, and − 7.6 kcal/mol, respectively, were selected as the more promising ligands. When compared with the standard drug tamoxifen (Ta) which binds to the active site of the macromolecule with ∆G_D_ value of − 6.3 kcal/mol, the promising ligands appear more potent than Ta in agreement with the results of *in vitro* studies. Similarly, the results of MM/GBSA calculations displayed in Table 3 revealed that the promising ligands bind to the receptor with binding Gibb’s free energy change (∆G_B_) that ranges from − 31.02 to − 40.37 kcal/mol. However, ligands 12, 16, and 21 which bind spontaneously to the receptor with ∆G_B_ value of − 40.37, − 40.31, and − 40.26 kcal/mol, respectively, appears to form more stable complexes with ER when compared with Ta which binds to the macromolecule with ∆G_B_ value of − 38.66 kcal/mol and as such they are chosen as the most promising ligands (MPLs).

Assessment of the mechanism of interaction of the MPLs with ER target shows that ligand 12 binds to the active sites of the receptor *via* two pi-alkyl interactions with LEU511 and LEU479; an unfavorable bump with ASN455; a π-donor hydrogen bond with THR 483; a conventional hydrogen bond with ARG515; and five Van der Waal interactions with GLU385, SER512, LEU508, ILE451, and ASP480 amino acid residues of receptor (Fig. 1). Also, ligand 16 forms three π-alkyl bonds with LEU511 and LEU479; an alkyl interaction with LEU508; three unfavorable bumps with ASN455; three π-donor hydrogen bonds with SER512 and SER456; one π-anion bond with ASP480; and seven Van der Waal interactions with THR483, TYR459, ARG515, GLU385, ILE452, ILE451, and HIS476 amino acid residues of the protein target (Fig. 2). Furthermore, the following interactions were observed in ligand 21/ER complex: a π-alkyl bond with ARG394; an alkyl bond with PRO324; a π-sulfur bond with TRP393; one π-sigma bond with ILE326; a π-anion bond with GLU323; three conventional bonds with TRP393, ARG394, and LYS449; a carbon-hydrogen bond with GLY390; and five Van der Waal interactions with MET396, GLU397, PRO406, LEU403, and GLU353 amino acid residues of the macromolecule (Fig. 3). The standard ER inhibitor (Ta) binds to the target through a л-л stacked T-shaped interactions with TRP 393; two carbon-hydrogen bonds with GLU323; five л-alkyl interactions with LEU403, PRO406, ILE 326, and ARG 394; an alkyl interaction with HIS398; and six Van der Waal bonds with MET 396, GLU 397, PHE445, VAL446, LEU320, and GLY442 amino acid side chain of ER (Fig. 4). In all the systems, hydrogen bond interactions which plays significant role in protein/ligand association were found except in Ta/ER system and this could be responsible for its relatively lower docking score. Absence of hydrogen bond in Ta/ER system was also observed by Maslikah et al., [37]. Summarily, hydrophobic, hydrogen bonds, and Van der Waal interactions are the major interactions in the complexes of the MPLs with ER target (Fig. 5). Hydrophobic interactions were highest in Ta/ER system, followed by 21/ER, 16/ER, and least in 12/ER. Also, the magnitude of Van der Waal interactions follows the order; 16/ER > Ta/ER > 12/ER, 21/ER. Furthermore, hydrogen bonds were highest in 21/ER and 16/ER, slightly high in 12/ER, and completely absent in Ta/ER system.

The ease of administration and high patient compliance rate that characterizes administration of drugs *via* oral route has made assessment of oral bioavailability a crucial component of modern drug discovery. The oral bioavailability profiles of the ligands presented in Table 4 reveals that all the ligands obey both Lipinski and Veber rules similar to the standard drug, indicating their high oral bioavailability potential.

Another crucial component of modern drug discovery is the prediction of the pharmacokinetic profiles of drug-like compounds in order to reduce potential attrition at the later stage of drug discovery. Pharmacokinetics is primarily concerned with how the body respond to the presence of a therapeutic compound in the body with particular emphasis on its absorption, distribution, metabolism, and excretion. The ADMET profiles of the ligands presented in Table 5 reveal that they have sound gastrointestinal absorption potential similar to the standard drug, Ta. Also, the promising ligands with the exception of 13 and 21 were found to be substrate of permeability-glycoprotein (P-gp), a membrane that prevents entry of toxins and xenobiotic into the body and maintains the integrity of the blood brain barrier [38]. The ability of the drug candidates to be adequately distributed in the body evaluated using the volume of distribution at steady state (VDss) reveals that all the ligands possess positive VDss values similar to Ta, indicating their likelihood of adequate distribution in the body. In addition, the entrance of chemical entities into the central nervous system is regulated by a membrane known as blood brain barrier (BBB). The pharmacokinetic profiles of the ligands reveal that they are substrates of BBB except ligands 14 and 21. Hence the ligands may not be neurotoxic unlike the two non-substrates. Furthermore, metabolism of the drug-like molecules in humans is performed majorly by two major isoforms of cytochrome-P450 enzymes known as CYP3A4 and CYP2D6. Similar to Ta, all the ligands were found to be substrate of CYP2D6 and as such could be effectively metabolized by this protein. Similarly, the ease of excretion of the therapeutic molecules evaluated using total clearance (TC) as a parameter reveals that the ligands have TC values that range from 4.68 to 9.26 log ml/min/kg and Ta has TC value of 10.36 log ml/min/kg. The positive values of this parameter in the drug-like molecules similar to that of the reference drug reveal their ease of excretion from the body.

In addition to having excellent anti-proliferative properties against MCF-7 cells, an ideal drug against breast cancer has to be of minimal or no toxicity. Thus, the toxicity profiles of the promising ligands (Table 6) reveal that ligands 4, 8, 12, 13, 16, and 21 have excellent toxicity profiles as they may not be mutagenic, carcinogenic, and non-biodegradable. Also, they may not cause liver injury or affects the reproductive system in any way. The standard drug, however was found to be carcinogenic and affect the reproductive system. This is particularly true as there abound reported cases of endometrial cancer and uterine malignancy associated with tamoxifen and other SERMs in ER+ breast cancer treatment [39], [40]. Furthermore, ligand 10 displays potentials for mutagenesis and reproductive deformities while ligand 14 may cause liver injury. Interestingly, all the MPLs have clean toxicity profiles as they are non-mutagenic, biodegradable, non-carcinogenic, and pose no threat of liver injury or damage to the reproductive system of man.

The coarse-grained based MD on the complexes of the three MPLs with ER are presented in Fig. 6. In essence, fluctuation of an amino residue in a protein-ligand complex is considered significant if its root mean square fluctuation (RMSF) value is > 2.5 Å [41]. In the complex of Ta with ER, fluctuations of the amino acid residues were below 2.5 Å *via* out the simulation trajectory except at 326 residue index (RI) where a major divergence of about 3.0 Å was observed. In 12/ER and 16/ER complexes, the amino acid residues of the receptor fluctuate below 2.5 Å throughout the simulation trajectories except for significant fluctuations of 5.6 and 3.0 Å, respectively at 330 RI. In ligand 21/ER complex, stable fluctuation of the amino acid residues was also observed along the simulation trajectory but significant fluctuations of 3.4 Å, 3.1, and 2.8 Å were observed at RIs of 330, 407, and 462, respectively. In all the four systems, equilibration occurs around 330 RI and the amino acid residues of ER fluctuates majorly at less than 2.5 Å through the trajectories, confirming the stability of the complexes as revealed by molecular docking studies and MM/GBSA calculations.

The frontier molecular orbitals made up of electron-rich highest occupied molecular orbital (HOMO) and electron deficient lowest unoccupied molecular orbitals provide information on the kinetic stabilities of molecules and the energies of these orbitals and other reactivity descriptors of the MPLs and Ta are shown in Table 7. The estimated energy gaps (∆E) in the MPLs were found to range from 3.66 to 3.78 eV and when compared with ∆E value of 4.51 in Ta, their ∆E values appear lower. Though the ∆E values in the ligands are high enough to confer some level of kinetic stabilities on them, the relatively higher anti-proliferative effects of the MPLs when compared with Ta could be partly attributed to their lower ∆E values as lesser ∆E value in a ligand has been linked with increased intra-molecular charge transfer culminating into higher binding interactions with protein target [44]. Also, lesser energy is required by the ligands to accept electrons than lose it as indicated by their lower electron affinity (A) than their ionization energy (I) values reflecting their possible electrophilic nature. The high values of ω in the ligands also tend to support their electron-loving nature. Furthermore, analysis of the DFT-derived molecular electrostatic potential (MEP) map (Fig. 7) of the MPLs and the standard drug reveals that the ligands have more reactive sites than Ta, and their higher spontaneous binding interactions with ER could be attributed to this.

## 5 Conclusion

In search of new drug candidates against estrogen receptor positive (ER+) breast cancer, bioactive colchicine analogues were screened against estrogen receptor using molecular docking techniques to identify the promising ligands. The outcome of the virtual screening revealed some new ligands with better binding interaction with the receptor than tamoxifen, a standard drug used herein for comparison. MM/GBSA studies was used to ascertain the influence of solvation on the binding interaction of the ligands with the receptor. The DFT (B3LYP/6-31G) method was used to investigate the kinetic stability of the most promising drug candidates while the time-dependent stability of the complexes of the most promising ligands with ER was established *via* coarse-grained molecular dynamic simulation. In addition, the identified drug candidates possess sound pharmacokinetic and toxicity profiles and as such could be novel sources of new drugs against ER+ breast carcinoma.

## Author contributions

RA and PJA outlined and designed the research work. RA and AS analyzed and supervised the study. PJA handled the computational chemistry software and drafted the manuscript. In addition, all authors read and approved the manuscript.

## CRediT authorship contribution statement

**Adnan Rohana:** Validation, Supervision, Conceptualization. **JOHN AMEJI:** Writing – original draft, Methodology, Conceptualization. **Shtaiwi Amneh:** Validation, Supervision.

## Declaration of Competing Interest

The authors declare that they have no known competing financial interests or personal relationships that could have appeared to influence the work reported in this paper.

## Data Availability

Data will be made available on request.
